# LiDAR Point Cloud Generation for SLAM Algorithm Evaluation

**DOI:** 10.3390/s21103313

**Published:** 2021-05-11

**Authors:** Łukasz Sobczak, Katarzyna Filus, Adam Domański, Joanna Domańska

**Affiliations:** 1 OBRUM Sp. z o.o., R&D Centre of Mechanical Appliances, Toszecka 102, 44-117 Gliwice, Poland; 2 Department of Distributed Systems and Informatic Devices, Faculty of Automatic Control, Electronics and Computer Science, Silesian University of Technology, Akademicka 16, 44-100 Gliwice, Poland; adam.domanski@polsl.pl; 3 Institute of Theoretical and Applied Informatics, Polish Academy of Sciences, Bałtycka 5, 44-100 Gliwice, Poland; kfilus@iitis.pl (K.F.); joanna@iitis.pl (J.D.)

**Keywords:** autonomous vehicles, LiDAR, autonomous driving, SLAM

## Abstract

With the emerging interest in the autonomous driving level at 4 and 5 comes a necessity to provide accurate and versatile frameworks to evaluate the algorithms used in autonomous vehicles. There is a clear gap in the field of autonomous driving simulators. It covers testing and parameter tuning of a key component of autonomous driving systems, SLAM, frameworks targeting off-road and safety-critical environments. It also includes taking into consideration the non-idealistic nature of the real-life sensors, associated phenomena and measurement errors. We created a LiDAR simulator that delivers accurate 3D point clouds in real time. The point clouds are generated based on the sensor placement and the LiDAR type that can be set using configurable parameters. We evaluate our solution based on comparison of the results using an actual device, Velodyne VLP-16, on real-life tracks and the corresponding simulations. We measure the error values obtained using Google Cartographer SLAM algorithm and the distance between the simulated and real point clouds to verify their accuracy. The results show that our simulation (which incorporates measurement errors and the rolling shutter effect) produces data that can successfully imitate the real-life point clouds. Due to dedicated mechanisms, it is compatible with the Robotic Operating System (ROS) and can be used interchangeably with data from actual sensors, which enables easy testing, SLAM algorithm parameter tuning and deployment.

## 1. Introduction

**Figure 1:**
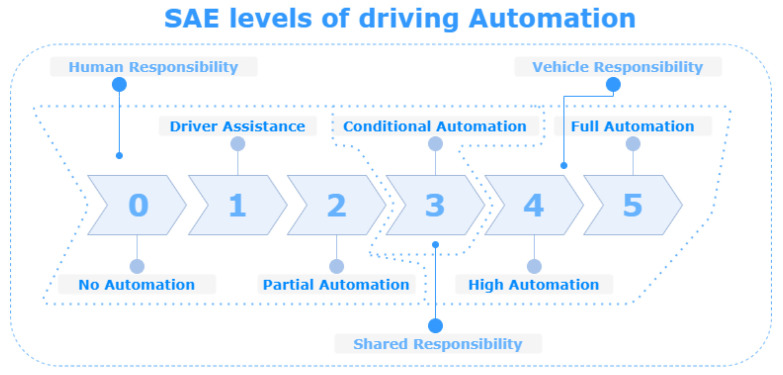
SAE levels of Driving Automation.

It is a fact that we face the beginning of a new mobility era. The automotive industry has put its interest in autonomous vehicles. The biggest technology companies such as Nvidia, Intel, Google and new mobility startups (Uber, Aurora and Cruise) are putting their effort into creating solutions for the autonomous car industry [1]. Autonomous driving on SAE level 4 and 5 (see Figure 1) has been classified as one of the biggest emerging technology trends in the Gartner Hype Cycle for Emerging Technologies 2019 [2]. Besides this glimpse of the future, in which we no longer need to drive our personal cars, there are much more critical areas in which reliable autonomous vehicles are vital. Here, we can mention safety-critical domains, e.g., mine detection, firefighting and contamination. Additionally, in other domains, which are simply not accessible to humans, e.g., Mars exploration.

Although the autonomous vehicles are considered an element shaping the future of mobility in BMW Group Safety Assessment Report [3], the idea to create self-driving vehicles reaches as far as to the first half of the 20th century [4]. At first, a lot of human supervision was necessary. For example, Stanford Cart (1961) was a remotely controlled cart equipped with a camera. They planned to use it on the moon surface and control it from the Earth using video transmission [5]. Ten to fifteen minutes were necessary to make a move, which was obviously too long for real-time operation. The 1980s brought two big projects (DAPRA–Autonomous Land Vehicle (ALV) [6] and the EUREKA–PROMETHEUS [7]). At that stage, autonomous vehicles’ requirements were defined: autonomous operation in a dynamic unconstrained environment, accurate environment mapping and track definition and maintenance [6]. Additionally, PROMETHEUS aimed to improve safety, and efficiency, as well as to decrease the pollution in everyday transport. During these times, first trials with artificial intelligence emerged, e.g., ALVINN (Autonomous Land Vehicle In a Neural Network) [8], which processed camera frames with a Multi-layer Perceptron and managed to complete a distance of 400 m autonomously, keeping its track. The new millennium brought autonomous driving to a new level. The race to create fully autonomous vehicles has taken the form of actual competitions. DARPA funded Grand Challenge Competition and Urban Challenge competitions. They included long stretches in difficult desert terrain and all the maneuvers common for the urban area with respect to the safety rules. DARPA competitions described above established the milestones in the area of self-driving cars and directed the automotive industry.

The autonomous vehicles are highly heterogeneous. They range from personal cars through rovers to tanks. Implementation of autonomous vehicles differs considerably for different sectors [9]. For example, vehicles operating in the commercial sector are easier to control due to the more inherent structure of the environment. In some of the applications (e.g., agriculture), additional structures can be added to improve the technology competency and to minimize the risk. On the other hand, military and safety-critical sectors can entail heterogeneous environments, often unstructured [9]. Here, a division of environments can be introduced: off-road/on-road or urban/rural/off-road. Obviously, operation in different conditions and terrains differs significantly and should be taken into consideration in the design process [10]. Vehicles can also operate on different levels of autonomy. These levels can be described using many different scales (e.g., SAE, classification used by US Navy Office of Naval Research or NATO Industrial Advisory Group [9], see Section 2.1).

To operate, autonomous systems use data gathered from multiple sensors, i.e., LiDARs (Light Detection And Ranging), cameras, RADARs, IMUs (Inertial Measurement Unit), and GNSS (Global Navigation Satellite System) (see Section 2.2). Data from these sensors are used in the four main components of the autonomous system, namely localization and mapping, understanding of the surrounding environment, path determination and vehicle control (see Section 2.4). The accuracy of the aforementioned sensors differs, e.g., camera data are highly sensitive to changes in lighting [11]. For that reason, for one of the most important and challenging fundamental tasks of autonomous driving, Simultaneous Localization and Mapping (SLAM) (see Section 2.5) data from LiDARs, is usually used. It is the most reliable data acquisition mechanism for this task [11]. Often, to perform the assigned tasks, approaches based on a fusion of multiple sensors are used, e.g., fusion of IMU and LiDARs in the Localization and Mapping task to improve the accuracy [12]. To deliver reliable autonomous solutions, testing of all kinds of autonomous driving algorithms is vital. Different testing approaches are possible: real-world testing, testing on real-world datasets, and via simulation [13]. Although the real-world testing and pre-recorded data can offer unexpected behaviors and diversity, conducting a sufficient number of experiments to cover all possible use cases and gathering large amounts of real-world data (which is vital for testing the autonomy algorithms) is very expensive and takes a lot of time [14]. Additionally, the labeling is non-trivial. Simulations, on the other hand, are much more flexible due to the feasibility of creating virtual worlds. It also seems to be the best approach for off-road and safety-critical domains testing because it does not require gathering real data from highly heterogeneous terrains and inaccessible environments.

The existing simulators, e.g., CARLA [15], AirSim [16] and LiDARsim [13] target testing of the following autonomous algorithms: driving in traffic jams, keeping the track while driving on roads, avoiding obstacles. Additionally, the papers regarding LiDAR Point Cloud simulation, e.g., [14,17,18], focus mainly on the generation of realistic scenes with the point cloud labeling for the purpose of object detection and recognition. A clear gap cap can be observed that covers the testing of autonomy algorithms operating directly on raw data, e.g., SLAM algorithms, which are considered to be one of the most important and challenging components of the autonomous systems [19]. Additionally, the existing approaches focus mainly on urban autonomous driving testing. They do not cover the off-road and safety-critical domains, where GPS data (the autonomous cars rely on it significantly in the localization process) is often not available, thus accurate and efficient operation of SLAM algorithms is critical. It is yet another gap in the area of autonomous driving simulations. Another inadequacy of the existing simulators is that they use simplified sensor physics and do not consider the true non-idealistic nature of the physical devices. The realistic character of data is vital for the purpose of accurate algorithm verification and for the process of algorithm parameters tuning.

In this work, we build a framework which can be used to evaluate the performance of fundamental autonomous driving algorithms operating directly on data gathered from sensors in real time, especially the SLAM algorithms. To create realistic and heterogeneous scenes (both the indoor and outdoor environments) and vehicles, we use Unity (mainly the manual modeling of scenes and vehicles). We also use the C# development environment to implement the realistic LiDAR behavior and the data collection procedure. We take into consideration measurement errors characteristic for the actual devices (in the paper, we use the term “noise”) and the so-called rolling shutter effect, which significantly impacts the quality and nature of the LiDAR data (described in more detail in Section 4.2). Additionally, we implemented additional mechanisms, which were necessary to create a realistic setup. We use a Robotic Operating System (ROS), which is a common system used in the area of robotics [20]. It delivers a set of tools and libraries (middleware), which facilitate software creation in the robotics industry. It imposes to use the Publish-Subscribe model of communication in the process of consecutive module design. It is also responsible for establishing the format of data transmitted between the system nodes. Additionally, it offers the tools which can be used to visualize data, e.g., laser scans or accelerometer reads. It can also be used in the process of development and debugging of the software elements created for the application in robotics. We use ROS to run the experiments with the SLAM algorithm of choice, Google Cartographer (3D), which is one of the leading SLAM algorithms (see the comparison of different SLAM algorithms in Section 2.5), which is also compatible with ROS. To connect ROS with Unity, we create dedicated scripts that wrap data from virtual sensors and send it via User Datagram Protocol (UDP) to a special ROS bridge. By doing so, data from virtual sensors is compatible with ROS and can be used interchangeably with data from actual sensors. This utility enables us to run our autonomous driving algorithms (e.g., SLAM) on ROS, which is installed on an actual device, which makes our approach more practical. After testing and tuning the parameters of our algorithm in simulation, we can easily switch to a real device operation. To evaluate the quality of obtained LiDAR point clouds, we compare the point clouds created using an actual device and a simulation reflecting the corresponding real-world environment. We also evaluate the performance of the simulation using Google Cartographer [21] SLAM algorithm (using different settings of the simulation), by comparing the errors obtained in an actual track and the corresponding simulation.

## 2. Background

In the following Section, we discuss vital aspects of autonomous systems. The aim is to describe the idea of autonomy, sensors, with which autonomous vehicles are usually equipped, the accuracy of the measurement equipment, main components of an autonomous system and their connection to the aforementioned sensors. As the article introduces a platform, which aims mainly at testing the key autonomous driving algorithms (but it is not limited to this task), especially Simultaneous Localization and Mapping (SLAM), we describe common algorithms (see Section 2.5), which can be used as a part of the Localization and Mapping component of the autonomous driving system. We use one of them, namely Google Cartographer, to verify our platform. We also describe different testing methods of autonomous vehicles to indicate the necessity of creating an accurate testing platform for autonomous systems.

### 2.1. Autonomy

A level of driving automation can be classified using levels defined by SAE International (a standard developing organization specializing in the automotive industry). Using the SAE levels, we can distinguish six levels of automation, which are illustrated in Figure 1. The SAE level 4 and 5 vehicles are high and fully autonomous cars, and they need a perfect understanding of the surrounding environment in real time. SAE is not the only classification that can be used. An early classification, introduced by Sheridan [22], distinguishes ten levels of automation, starting from no assistance, in which the user is responsible for all actions, to complete autonomy of the computer, which ignores human. US Navy Office of Naval Research uses a similar classification, which consists of six levels describing vehicles: human-operated, human-assisted, human-delegated, human-supervised, mixed initiative and fully autonomous [9]. NATO Industrial Advisory Group identified four levels of autonomy based on different industries view on autonomy degree and using the Observe, Orient, Decide, Act (OODA) loop: remotely controlled system, automated system, autonomous non-learning system, and autonomous learning system [9]. Other autonomy assessment scales (multi-dimensional) were introduced by Proud et al. [23] (8 levels of automation with description regarding all stages of the OODA loop per each level), and Clough [24] (11 levels, also described in accordance with OODA loop).

### 2.2. Equipment of Autonomous Vehicles

To deploy an autonomous vehicle, in the design process, four main components must be created, responsible for localization and mapping, understanding of the surrounding environment, determination of path, and vehicle control. All of them use data gathered from different sensors with which an autonomous vehicle is equipped. Sensors can be divided into two groups: active and passive. Active sensors make the measurements based on the reflection of the signal they sent. Unlike the active sensors, passive sensors use signals already existing in the environment (e.g., light or energy) [25]. The whole system should operate in various weather and light conditions. To maximize the effectiveness of an autonomous system, multiple sensors can be used, namely LiDARs, cameras, RADARs, IMUs, and GNSS. LiDAR is a device that emits laser beams and precisely measures the time necessary for the reflected beam to reach a photodetector. Usually, lasers with a wavelength of 905 nm are used. Common 3D LiDARs are equipped with 16 to 128 lasers arranged vertically and rotating with a speed of 1200 rpm at max. Data gathered in this process is used to create a point cloud, which can be used to map the environment and for positioning of the vehicle [26].

### 2.3. Accuracy of Sensors

All the aforementioned sensors are used by the autonomous driving algorithms (e.g., SLAM, object recognition). However, they exhibit some limitations, especially the camera-based approaches [27]. The advantages of using LiDARs are: higher resolution and broader field of view than these in RADARs and ultrasonic sensors and robustness under different conditions, including operation in light and dark environments, as well as with and without shadows and glare [14]. In opposite to camera-based sensors, LiDARs provide more accurate, robust, less noisy and sensitive to changes in the lighting, and for that reason, it is the most reliable mechanism of data acquisition for the SLAM algorithm input [11]. Therefore, it is of great importance to deliver accurate models of LiDARs to enable the generation of realistic data to test and verify novel SLAM algorithms. Nevertheless, it is important to remember that the real world is far from idealistic. The gathered LiDAR data still contains some noise. LiDAR data are subject to errors caused by the impact of the target color and material, atmospheric correction, beam divergence, target size and instrumental errors. Therefore, it is crucial to verify novel algorithms (especially those operating on raw LiDAR data, e.g., SLAM) using accurate, non-idealistic and demanding data (real or simulated). Gathering large amounts of heterogeneous actual data from different environments (especially those off-road and not accessible to humans) is impractical (and often impossible). That is why it is crucial to build simulation frameworks, which could be used to test and verify SLAM algorithms, especially in difficult terrains.

### 2.4. Components of Autonomous Driving System

Four main components are vital for the autonomous system: localization and mapping, understanding of the surrounding environment, determination of a path, and vehicle control.

Localization and mapping can be divided into global and local ones. Global Localization and Mapping is usually based on Global Navigation Satellite System (GNSS) to determine the position of the vehicle with respect to the globe. GPS data are not always available (due to limitations in the access to satellites) and even if it is accessible, it is a subject to measurement errors. To improve the accuracy of the algorithms or to enable the operation when GPS signal cannot be used, data from other sensors can be used (usually with IMU data and odometry sensors). Global localization is vital for determining the approximate position of the vehicle. However, it can be insufficient for more complex operations (e.g., keeping the vehicle on the determined track), for which more precision is necessary. Here, a local system is used. Based on the input data (usually from LiDARs and cameras), it creates a model of the surrounding, and then, using the Simultaneous Localization and Mapping (SLAM) algorithms, enables localization of the vehicle in the obtained model of the environment. Sensor data fusion can also improve the precision of the localization and mapping algorithms [28].

Understanding of the surrounding environment can be divided into three main subtasks: object segmentation, detection, and classification. Here, a model of the environment obtained in the Localization and Mapping process is examined to interpret the surrounding environment, which includes static and dynamic objects. For that purpose, data from different sensors are used, usually LiDARs, cameras and RADARs. Semantic segmentation aims to categorize every pixel in the gathered data [29]. Deep Learning and Convolutional Neural Networks have proven to be successful in this area [30,31]. The next steps are detection and classification of the particular segments (objects) of the environment. For dynamic objects, RADARs data are used to determine the speed of the object. Additionally, for this task, the most popular approaches are based on Deep Learning [32,33]. Sensor data fusion allows achieving a better data representation, thus more accurate classification [34]. Detection, localization and classification of obstacles enable the creation of occupancy grids containing information on the environment. Additionally, a determined class of the object, its properties, e.g., speed, can be used to forecast its future behavior.

Path determination requires knowledge about the vehicle location, destination and a complete understanding of the surroundings. The main target of this task is to ensure successful and safe departure to the destination. Two types of path determination can be differentiated: local and global ones. When the environment is known, global planning aims to determine the best track (e.g., in terms of time, distance, safety regarding a chosen cost function). Graphs are useful in determining such a path [35]. In the case of an unstructured environment, global planning limits to determine the path in a correct direction based on the information about the already completed fragments of the track. Local path determination uses algorithms such as A* [36] or D* [37]. Other methods have also been developed for off-road driving [38,39].

The last autonomous driving component, vehicle control, is responsible for sending control commands to the vehicle’s subsystems to enable its appropriate driving (acceleration, braking, wheel steering angle).

In the design process of an autonomous system, it is crucial to use an accurate dynamic model of the vehicle [40]. Besides that, to establish the optimal set and arrangement of sensors, design the proper autonomous driving algorithms and tune their parameters, and eliminate potential flows before the deployment, testing is vital.

### 2.5. 3D SLAM Algorithms

SLAM is one of the fundamental and the most challenging autonomous driving algorithms [19]. Different types of SLAM algorithms can be used for Localization and Mapping. They can use data from different sensors, usually cameras or LiDARs. As mentioned before, LiDARs provide data that is more accurate, robust, less noisy, and sensitive to changes in the lighting. For that reason, it is the most reliable mechanism of data acquisition for the SLAM algorithm input [11]. Some approaches use a fusion of laser and visual data [41]. These algorithms, depending on a type, can operate on 2-dimensional [12,42,43] data or 3-dimensional [44,45] data (or both [21]). In the case of LiDAR-based SLAM, the algorithm takes as an input LiDAR point clouds. Two-dimensional LiDAR-based SLAMs are more computationally efficient than 3D ones. However, due to the limited number of dimensions, they are not able to provide the estimation of the vehicle’s pose on uneven ground with six degrees of freedom (DOF) [46]. Additionally, with the limited number of features, it is hard for this type of algorithm to adapt to long laneways, which are highly similar. Different methods to restore 3D information from 2D point clouds can be used. Nevertheless, it makes the configuration complicated [46]. Therefore, in this work, we focus on testing 3D SLAM algorithms, which provide a more accurate estimation of the environment.

Google Cartographer [12,21] is a SLAM algorithm that can operate on 2-dimensional and 3-dimensional LiDAR data. Cartographer uses two types of SLAM: the local one to create sub-maps and current trajectory and the global one, which matches these sub-maps and creates a global map [47]. This mechanism is used to detect loop closure, which is a common task in the autonomous driving domain. Global optimization is responsible for the correction of the global map in the case of loop closure detection [48]. The main purpose of the algorithm is to calculate the coordinates of the vehicle, determine the surrounding environment and send it to a navigation algorithm. Cartographer takes small data packets as input and uses them to create a full 3D scan by calculating the translation of the individual parts of this scan (to minimize the impact of the rolling shutter effect, described in Section 4.2). Here, the scan matcher is penalized for matching the scans in the way that they highly deviate from the prior position (it is assessed by a special score). Penalty refers to deviations in rotation and translation. Weights of this penalty are examples of the parameters that must be tuned for different conditions and environments. Accurate sub-maps are vital for global optimization, thus the creation of reliable maps. Google Cartographer can use data from different sensors and functionalities (e.g., GPS) and allows the user to supplement it with additional information sent in a proper format to improve the localization and mapping process. It is also easy to integrate it into ROS [20].

HDL Graph SLAM is similar to Cartographer in some respects since both approaches use Graph SLAM. The most important difference is that HDL does not use IMU data to estimate odometry but instead calculates it based on the LiDAR data. Based on the Figures presented in [49], it can be observed that the accuracy of HDL SLAM degrades with time, so it is unpractical to use it for long paths in the unknown environment. They also claim that they were unable to obtain a coherent model of the environment using this SLAM algorithm on the test dataset. Additionally, HDL SLAM operates only using 3D point clouds. In contrast, Cartographer can operate on both 2D and 3D data. In [50], it was proven that Google Cartographer outperforms other algorithms taken into consideration.

Other 3D SLAM algorithms include LiDAR odometry and mapping (LOAM) and normal distribution transform (NDT) SLAM. In [51], it was proven that Google Cartographer outperforms them in terms of efficiency. RTAB (Real-Time Appearance-Based) SLAM algorithm, on the other hand, strongly depends on data from cameras, which is used to deal with loop closure effect [52].

Google Cartographer is one of the most popular SLAM algorithm in recent research works, therefore, it can be treated as the state-of-the-art SLAM algorithm. Thus, it is reasonable to verify the suitability of our simulation in terms of modern SLAM algorithms testing.

### 2.6. Testing of Autonomous Vehicles

To verify the performance of autonomous vehicles, different testing approaches can be used: real-world testing, testing on real-world datasets, and via simulation [13]. There is no perfect solution for testing, because all the approaches have their advantages, but also constraints. Real-world testing can offer unexpected behaviors and diversity but can be a danger to safety (especially in urban environments). It is usually performed in controlled environments. Testing on pre-recorded data also offers diversity but requires gathering large amounts of data and exhibits the limits of real-world testing. Both approaches are constrained by a limited number of use cases because such a testing can be very time-consuming and costly. Additionally, the labeling (for segmentation and object recognition tasks) of large amounts of real-world point clouds is very expensive and takes a lot of time [14]. Real-world testing entails logistical difficulties, high infrastructure costs and makes it impossible to conduct a statistically significant number of test cases [53]. Simulations, on the other hand, are much more flexible because of the feasibility of creating virtual worlds. This approach seems to be the best one, especially for the off-road and safety-critical domains testing, because it does not require gathering real data from the highly heterogeneous terrains and inaccessible environments.

## 3. Related Work

Due to the emerging interest in the area of self-driving vehicles, more and more focus is put on the simulation and generation of accurate and realistic data connected to autonomous vehicles. Many solutions from this area are based on machine learning algorithms (especially neural networks and Deep Learning), for which it is necessary to provide large amounts of data for effective training. Most works, which analyze information included in LiDAR point clouds are focused on object segmentation, classification, and simulation of vehicle behavior during different circumstances [54,55,56,57]. Therefore, also the simulators themselves are focused mainly on accurate object generation and not the accurate reflection of the nature of the point clouds (noise caused by measurement errors and phenomena characteristic for this type of data, e.g., the rolling shutter effect).

The papers regarding the simulation frameworks for the LiDAR Point Cloud generation include [13,14,17,18,53]. In [14] they present a framework, which can generate a point cloud with point-level labels using a simulation based on an auto-driving computer game and manually configured scenes. They also used an automatic calibration method to mark the point clouds on the corresponding scene images. The main target of this research was to create a framework that can generate large amounts of annotated data for the purpose of neural network training (especially for segmentation tasks). In [53] they proposed the physics-based simulator of a LiDAR, targeting the highly vegetated environments. The aim of the framework was to test off-road autonomous navigation in complex outdoor environments by capturing the interaction of a laser beam with plants. Paper [17] presents a simulation framework using a real environment and real traffic flows. They use an actual LiDAR to obtain the real-world scenes and use these images in the simulation framework. They remove the moving objects (e.g., cars and pedestrians) from scenes and enrich them with synthetically generated obstacles. From these augmented scenes, new point clouds are generated. Such obtained data can be used to improve the accuracy of the object detection capabilities of an autonomous vehicle. In [18] they present a solution for synthetic LiDAR Point Cloud generation with the automatic ground truth annotation. In the framework, both 2D and 3D sensors can be simulated. They test the quality of the generated data on a semantic segmentation task and present the comparative results with other methods.

The advancements in rendering techniques allowed for the creation of autonomous driving simulators, e.g., CARLA and AirSim [13]. In these efforts, there is a clear gap for the simulation targeting the evaluation of the algorithms based on autonomous systems (e.g., SLAM). The simulators above aim to test mostly algorithms such as keeping track while driving on roads, driving in traffic jams, avoiding obstacles, etc. Airsim can be used as a Unity plug-in; however, the current release is only experimental. Additionally, the approach has the same limits as AirSim run on the Unreal engine (the original one). In contrast to these frameworks, our simulator aims at testing even those algorithms which are at the basis of autonomous systems (and this is what we put emphasis on), such as SLAM, processing of LiDAR data (point clouds), processing of IMU data (Kalman filter), creation of obstacle map (so-called occupancy grid), object detection, semantic segmentation. CARLA and AirSim are also simplifying the physics assumptions and do not reflect accurately noisy and distorted data from real-world sensors [13]. Other simulators, such as LiDARsim [13], use real-world data to create realistic scenarios, which is good for urban environments, but not practical and sometimes impossible for the off-road and dangerous environments with a virtually infinite number of use cases, terrain types and obstacles. In the paper, they also add the additional noise to data (via Artificial Neural Networks) to make it more realistic, but this approach may be too computationally demanding and time-consuming for a real-time simulation. Therefore, in our work, we chose random errors in the range of accuracy of measurements of the LiDAR sensor. All of the aforementioned simulators put focus on urban driving simulation, but what is of even greater importance it is to deliver a simulation which will enable testing of autonomous vehicles in highly heterogeneous environments, where there is no access for human beings (e.g., space exploration) or this access is highly limited or dangerous (e.g., minefields, firefighting). Therefore, in our work, we focus on the creation of a simulation, which will enable testing, especially in safety-critical domains. We put special attention to make the data generated by our LiDAR simulation as realistic as possible by adding noise and taking into consideration the phenomenon, which significantly impacts the performance of the autonomous driving algorithms (rolling shutter effect, described in Section 4.2). We also implement additional mechanisms to make the data obtained from our simulation compatible with the autonomous driving software run on Robotic Operating System installed on an actual device.

## 4. Methodology

In this Section, we describe the technologies and the setup used to create our simulation and to verify the obtained results. We describe how we use particular technological components, namely Unity, ROS and additional software. We discuss the importance of the rolling shutter effect and the method we use to implement it in our simulation. We also present the parameters that can be used to control the simulation. We describe the process of data collection and two types of evaluation of our framework: Point Cloud Comparison-based and SLAM-based ones.

### 4.1. Experimental Setup

**Figure 2:**
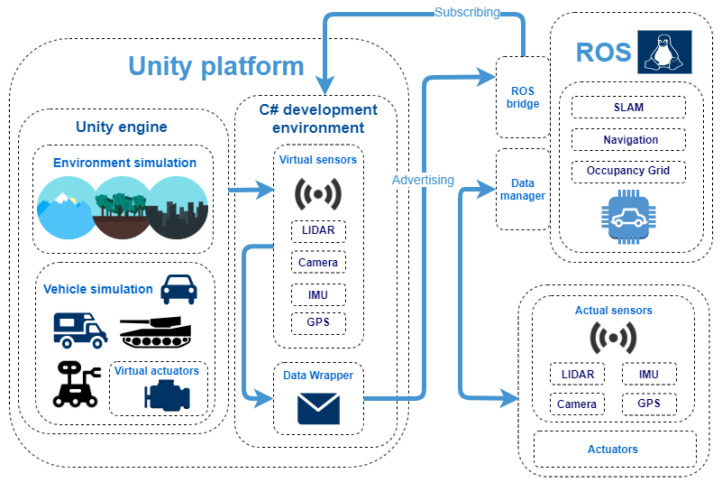
Experimental setup.

In our experiments, we use three main components: Unity platform or actual autonomous vehicle, Robotic Operating System and additional scripts to enable the communication between the components. All the components and the connections between them can be observed in Figure 2.

Unity is an Integrated Development Environment (IDE), which delivers a cross-platform graphics engine and can be used to create realistic 2D and 3D computer games, visualizations, and other interactive elements. To develop such an element, ready-to use components from the unity asset shop can be used to build the scenery. The platform enables the user to implement C# programs, which can modify the virtual scenes, objects, lighting, audio and other elements. Using the Nvidia PhysX physics engine, users can define the physical properties of objects (e.g., mass, velocity, acceleration) and define interactions between them (e.g., friction) and the environment (e.g., gravity). We use these components to create the realistic scenes of the simulation and create the virtual models of sensors (LiDAR, IMU, GPS and wheel encoder), which are then programmed by implementing the C# scripts. Data gathered using these sensors can be used by the various algorithms: responsible for mapping and localization (SLAM), path planning (Dijkstra’s algorithm [58]) and navigation (occupancy grid [59]). In the process of creating new algorithms, one of the most important stages is validation. To evaluate the efficiency and performance of the algorithm under observation, one can do it in a real-life environment, though this solution is very costly. As an alternative, it is necessary to perform this task in a virtual environment, which accurately simulates the real one. To achieve this, the test maps were created, mimicking a real-life space and labyrinths. We also built off-road environment maps, which allow us to validate the performance of the SLAM algorithms in external conditions. Additionally, the geometric and physical models of real-life vehicles were created to test the autonomy systems. To enhance the simulation functionality, a *bridge* between the simulation environment and ROS was created.

### 4.2. Simulation of the Rolling Shutter Effect

**Figure 3:**
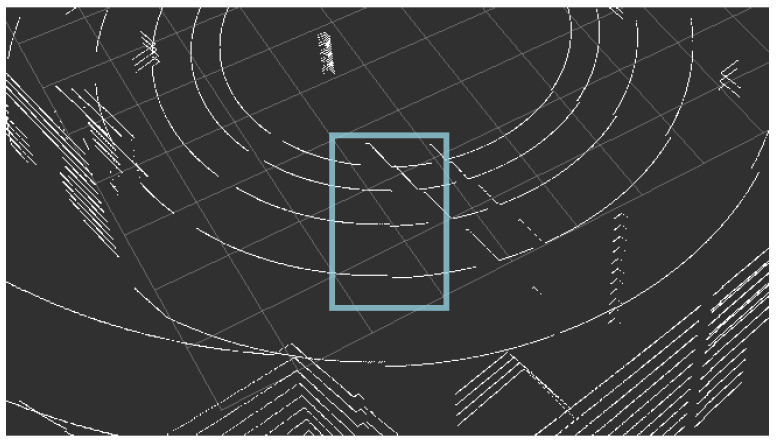
A slight effect of the rolling shutter effect observed in the point cloud obtained from an actual device (Velodyne VLP-16) driving around the laboratory.

The term of the *rolling shutter effect* [60] is usually used in the field of photography to describe the characteristic distortion on the pictures of the quickly moving objects, for which the movement occurs perpendicularly to the camera axis. It is caused by the construction of the shutter mechanism in the cameras and the line-by-line retrieval of the images from the photosensitive matrix. A very similar effect can be observed in the LiDAR context [61,62], though the origin, in this case, is different. The data are gathered using a laser beam or multiple laser beams distributed vertically and a detector. This equipment set rotates (usually 20 rps at most) and delivers the data which is used to estimate the distance. Due to the relatively small spinning frequency of LiDARs, the time needed to acquire a single scan is relatively long (e.g., 10 Hz for Velodyne VLP-16 spin frequency translates into 100 ms duration of a full scan). If the translational motion speed is high in comparison (a significant movement of the vehicle is observed) to the rotational movement, the distortions in the gathered point cloud occurs, which is caused by the translations during the full rotation. The Velodyne LiDARs deliver the data in small packets, in which the distortion rate is low, but in the full view, on the borders of the subsequent fields, the effect is clearly visible. Due to these distortions, it is not possible to directly (rigidly) transform the packets of the frame, but a continuous transformation or its approximation is necessary (or some other technique to minimize this effect should be applied by a SLAM algorithm) [61]. Laser scan measurements are often subject to the rolling shutter effect in the case of the significant movement of the vehicle and not taking this effect into consideration by a SLAM algorithm can result in serious map quality degradation and pose estimation errors [62]. Figure 3 presents a slight rolling shutter effect. It is hard to observe a very visible effect in a relatively small environment because the space is limited and a high speed cannot be achieved, but even in such an environment, this effect can be encountered and should be taken into consideration. In the simulation of the outside environment, where space is not limited and high speed can be easily achieved, the impact should not be omitted if the target is to obtain an accurate reflection of the real-life behavior.

To realistically simulate the LiDAR-obtained point clouds in the simulation, it is important to include the simulation of the rolling shutter effect. With each update of the physics engine, the packets with a minimal portion of data are generated, which is then used to simulate the effect. This approach is particularly important for the simulation, which aims to be used for the purpose of validation of new SLAM algorithms. The SLAM algorithm used in the experiments, Google Cartographer [12], uses small portions of data obtained from LiDAR and accelerometer readings to estimate the vehicle movement and to adjust the subsequent data. As a result, a more accurate estimation of the rotation and translation during the SLAM algorithm run-time can be achieved.

### 4.3. Collection of Data Points

To accurately simulate an actual electronic device, it is crucial to know its construction and the method of data acquisition used. Our implementation of the LiDAR simulation allows us to define many parameters of a typical LiDAR: any number of laser beams and any viewing angle, both the vertical and the horizontal ones, also the frequency of rotations per second and the maximum error of a single distance measurement. The values of the parameters set have been presented in Table 1. The parameter Mode in the LiDAR context means that either the strongest beams reflected, or the last ones are taken into consideration in the process of gathering data for each channel. In the simulation, we decided to use the strongest beams, because these beams are the most significant and stable ones, therefore more suitable for the real-time simulation and the SLAM algorithm input. We use a higher Horizontal Field of View (FoV) per simulation update to accelerate the processing and enable the real-time simulation. Additionally, an additional parameter can be defined, concerning the rolling shutter effect. It defines the size of a point cloud data slice imitating a scan of an actual, imperfect device. It is important to properly place the device in the space surrounding the vehicle. It is obtained by definition of the positions with respect to previously prepared places on a virtual 3D model.

The operation of a virtual sensor (in this case, LiDAR) is strongly dependent on the length of a physics simulation step. Distance measurements to specific points in space are performed depending on the data delivery frequency and take place once in a simulation update at maximum. To obtain the measurements, we use ray-tracing technology. Laser beams are emitted in parallel and then return to a device. The measured distance is a distance to the nearest obstacle in a virtual environment. Because these measurements take place in a time that is usually shorter than the duration of the physics update, a full 360 deg scan does not contain the rolling shutter effect discussed earlier. Therefore, the appropriate parameterization of a LiDAR model allows us to provide information about the shifts of individual scan sections to mimic a fast vehicle movement when it is necessary.

### 4.4. Evaluation Methodology

**Figure 4:**
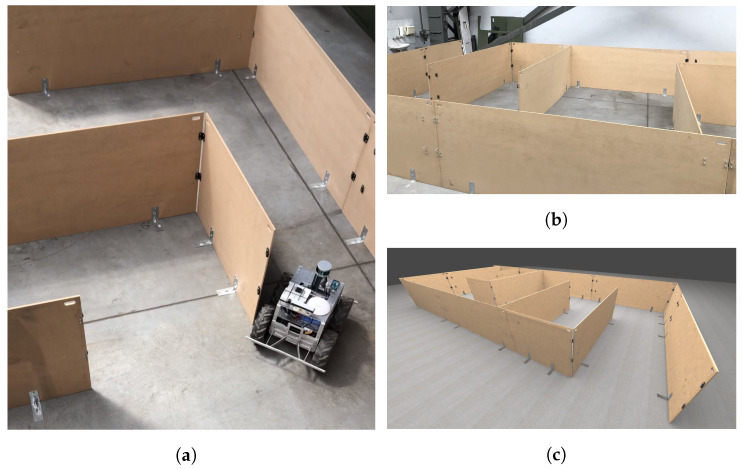
A real-world track (labyrinth) built for the purpose of the evaluation and the corresponding simulation. (**a**) A photo of an actual track; (**b**) A photo of an actual track; (**c**) A screenshot of a simulated track.

We evaluate the framework by comparing the results obtained using point clouds obtained from an actual device and a corresponding simulation. In Figure 4 we present the evaluation setup. We use two methods to generate the data: with and without the generated noise. The evaluation consists of two stages. First, we compare the accuracy of an artificial point cloud simulation by measuring the distance of the simulated and real-world measurements (see Section 4.4.1). The second stage is based on the examination of SLAM performance metrics obtained from running an algorithm on real-world data and the simulated one (see Section 4.4.2).

#### 4.4.1. Point Cloud Comparison

**Figure 5:**
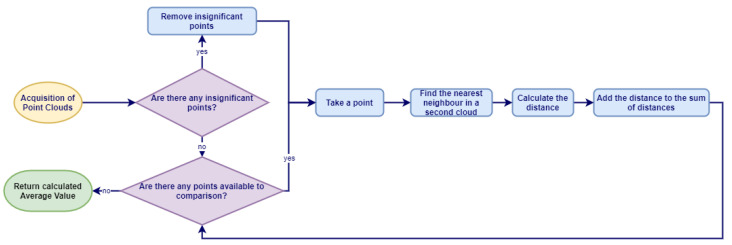
The algorithm used to evaluate the distance between the point clouds.

To compare two point clouds and evaluate the accuracy of the simulation, we propose an algorithm which is presented in Figure 5. Point clouds are compared at six characteristic checkpoints marked by a measuring tape and projected to simulation. First, we remove the insignificant points, e.g., in the examined case, all the points above the height of the labyrinth. Depending on the point cloud, this can accelerate the algorithm by several percent. Afterward, we iterate through the points, and for every point, we find the nearest point in the second one and sum up a distance between these two corresponding points. We repeat the same procedure using the second point cloud as the reference. For every cloud, we calculate the average distance between the points, and then the average of these values. The obtained value is the final score which describes the average distance between the point clouds.

#### 4.4.2. SLAM-Based Evaluation

**Figure 6:**
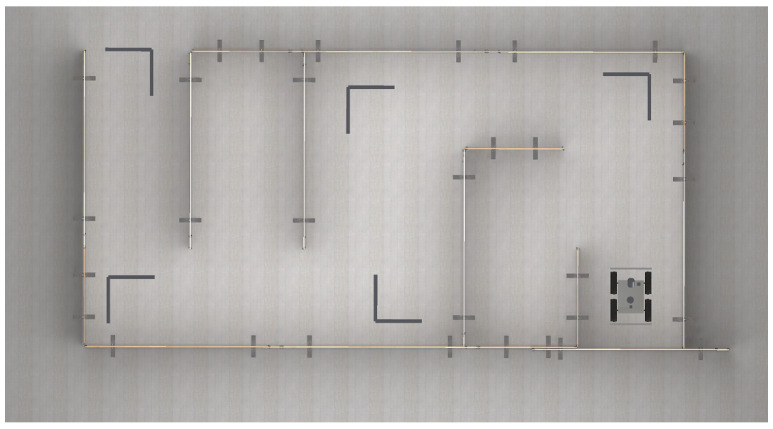
Track no. 2: A labyrinth setup with 5 checkpoints and a start line used for the evaluation.

We conduct the evaluation using SLAM algorithm provided by Google Cartographer. To the algorithm, we input point clouds generated using an actual vehicle equipped in LiDAR and IMU and a simulated point cloud and inertial data with or without noise. We perform experiments ten times, and we average the results for each of two test tracks, which we also mapped in the virtual environment:

- *Track no. 1*—drive over a straight 4-meter section with 4 measuring points (the first one is also taken into consideration, so we have 5 measurement points in total) once in every 1 m in the test room,
- *Track no. 2*—a labyrinth with 5 checkpoints and a start line (Figure 6).

For every estimated pose *p* we also have the reference pose $p^{\prime }$ and we can calculate a sum of squared distances between estimated and ground truth poses in moments 1 to *N* using the following error function [63]:

(1) $$\epsilon \left(p_{1:N}\right)=\sum_{i=1}^{N}{\left(p_{i}⊖p_{i}^{\prime }\right)}^{2}$$

The authors in [63] claim that the metric could be suboptimal for comparing the result of SLAM algorithms. It is also a good manner to compare the performance of the same algorithm on data from different sources. In our case, the error results should be as similar as possible to the real ones because when these values are too low, the data are too idealistic and opposite—too noisy—and as a result, the evaluation achieved in both cases is not accurate. In other words, our aim is to generate the data on which a particular SLAM algorithm performs in the most similar way as on the real-life data, and we evaluate it using the error values for different measurement points.

## 5. Experiments and Results

**Figure 7:**
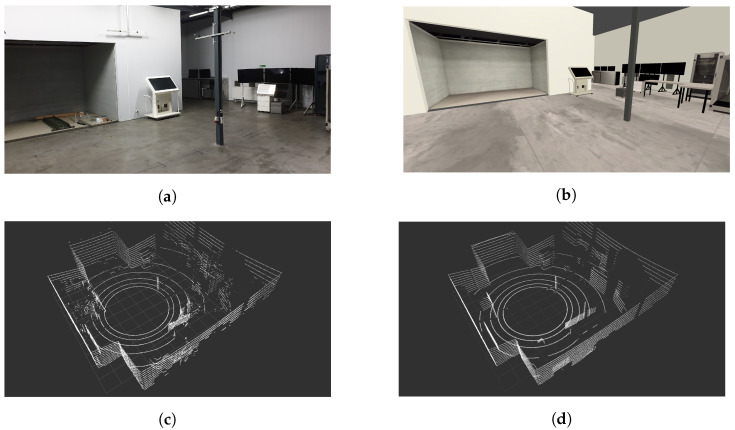
Sample LiDAR Point Cloud extracted from a real device in a real-world observation versus obtained from a simulation framework. (**a**) An actual photo of a laboratory room; (**b**) A screenshot of a simulated laboratory room in Unity; (**c**) Extracted point cloud from a real device—Velodyne VLP-16; (**d**) Extracted point cloud from a simulation.

**Figure 8:**
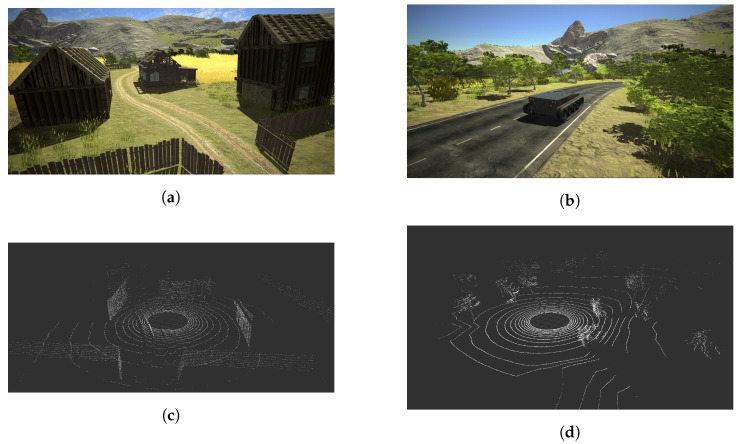
Sample LiDAR Point Cloud extracted from two simulated heterogeneous environments. (**a**) A screenshot of a simulated rural environment; (**b**) A screenshot of a simulated natural environment; (**c**) Extracted point cloud from a simulated environment (**a**); (**d**) Extracted point cloud from a simulated environment (**b**).

The capabilities of the framework are presented in Figure 7 and Figure 8. Figure 7 shows the actual physical laboratory with the corresponding simulated one and the point clouds obtained for both. Figure 8, on the other hand, shows two simulated outdoor environments with the corresponding point clouds.

The experiments were conducted using a 16-channel Velodyne 3D LiDAR with a horizontal and vertical field of view of 360 deg and 30 deg respectively, the full scan frequency of 10 Hz. We built the corresponding simulation model. We arranged the point clouds obtained in the marked points both in the simulation and the real-life setup. We consider the simulation with the additional noise and without it. The interferences are adjusted to the declared accuracy of measurements.

**Figure 9:**
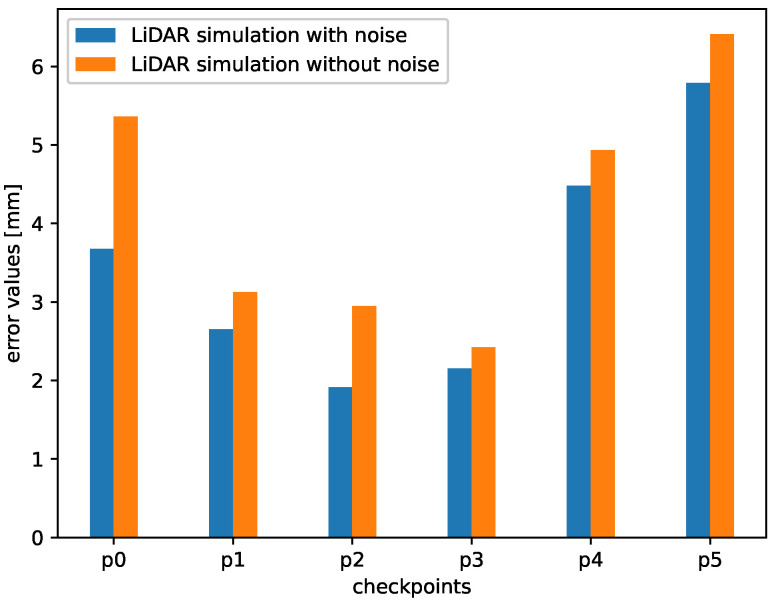
Mean error values between points from real and simulated point clouds.

In Figure 9, we present the average error value between the subsequent points in the clouds obtained from an actual device and a simulation. We use the track presented in Figure 4. We can notice that the errors for both simulated data with and without noise are low, approximately 6 mm and 6.5 mm, respectively, which means that the similarity in terms of a distance between the points of the simulated and real point clouds is high. We can clearly see that adding noise to the simulated point cloud decreases the mean error values for all the checkpoints and additionally increases the similarity.

**Figure 10:**
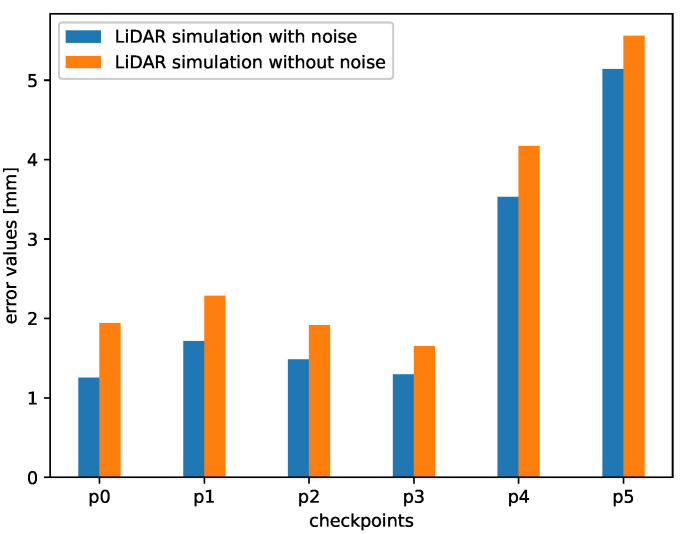
Mean error values between points from real and simulated point clouds (after alignment).

A significant part of the error value is caused by the imperfect placement of the vehicle in the actual labyrinth and the simulated one. Obviously, this error can be reduced by a proper alignment of the point clouds. For that purpose, we can use manual alignment or, as an alternative, the Iterative Closest Point (ICP) algorithm. ICP algorithm is a well-known technique to align the three-dimensional models when their initial position is given [64]. In our experiments, we decided to use manual alignment. In Figure 10 we can see that the error values are lower than in the case without the alignment. The reason is that we reduced the error caused by the small shifts in real and simulated environments. Nevertheless, even without this alignment, which can be difficult for practical applications, the point clouds in all the checkpoints are affected by an error of only a few millimeters because of the lack of alignment.

Comparing both Figure 9 and Figure 10, we can notice that the error bars for the checkpoints 4 and 5 were not so affected by this operation. It is because these checkpoints were placed at the end of the labyrinth. As we can see in Figure 4, in these measurement point, the labyrinth is opened, and the background is reachable for the LiDAR beams. We can also notice that in the real-life setup there are some objects in the room, a wall etc. and the simulated environment is empty. Our goal was to simulate the track, therefore we decided not to model the surroundings.

**Figure 11:**
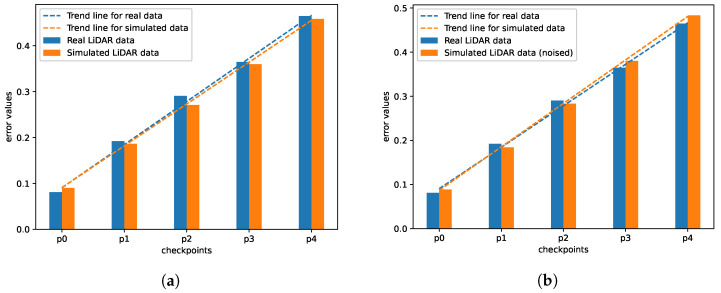
SLAM accuracy metric for real data and simulated data on the first track: (**a**) without noise and with the rolling shutter effect (**b**) with added noise and the rolling shutter effect, distance between the checkpoints—1 m.

**Figure 12:**
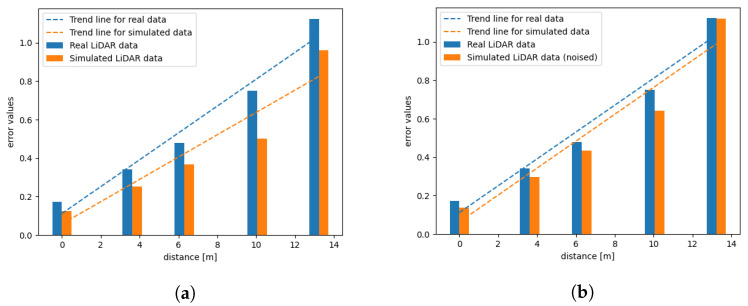
SLAM accuracy metric for real data and simulated data generated for the second track: (**a**) without noise and with the rolling shutter effect (**b**) with added noise and the rolling shutter effect.

According to the methodology described in Section 4, the SLAM accuracy tests have been performed using the data obtained from two different types of test tracks. Additionally, we aim to compare the results obtained from the simulation with and without the noise included. To add the proper amount of noise to the data, we based this amount on the information on the measurement error given by the Velodyne. The results can be observed in Figure 11 and Figure 12. We can observe that creating a realistic and detailed setup makes the simulated data similar to the corresponding real data.

In Figure 11 we can observe the error results achieved using Track no. 1. The error results of real and simulated data obtained using this track, which was a relatively simple track—a straight line with five measuring points—are similar (both for the simulated data with and without noise). In Figure 11a, we can notice that for the simulated data without noise added, it is the error on real data which increases faster, and in Figure 11b it is the opposite: the error is growing faster for the simulated data. However, it is only visible for the two last points. For this case, the SLAM algorithm behaves similarly on simulated data as on the real one. Therefore, both point clouds are suited to evaluate a SLAM algorithm.

**Figure 13:**
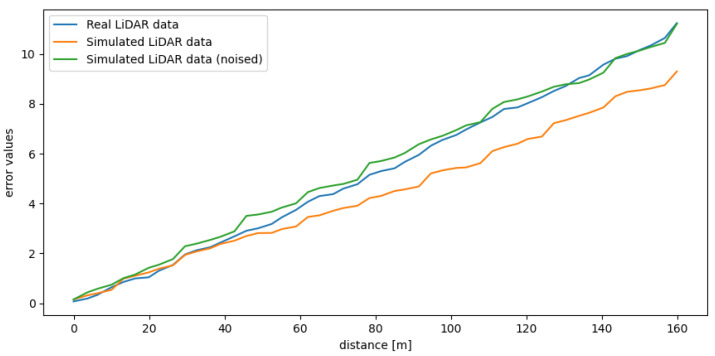
SLAM accuracy metric for real data and simulated data generated for the second track (obtained on this track for a vehicle travelling back and forth along this route).

In Figure 12 we can observe the error results achieved using Track no. 2. In this case, the differences in the errors obtained from the simulated point clouds with and without noise in comparison to the ones obtained from real data differ significantly. Here, we examine a more complicated track. In Figure 12a we can clearly see that the difference between the trends of both errors increases with time. If we wanted to use a longer track, the generated point clouds could not be accurate enough to evaluate the performance of the SLAM algorithm. Additionally, see Figure 13 to observe that the relationship between the accuracy metric and the distance from the beginning persists.

**Figure 14:**
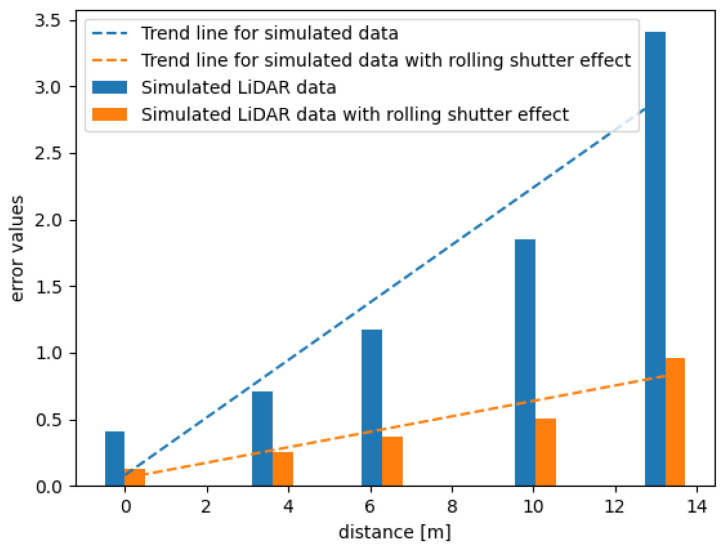
SLAM accuracy metric for simulated data generated for the second track with and without the rolling shutter effect.

Moreover, the difference between the errors obtained using simulated data with and without the rolling shutter effect increases significantly with time. It can be observed in Figure 14. It is not possible to match the linear trend well to the bars representing data without the rolling shutter, as the error increase is close to the exponential one.

**Figure 15:**
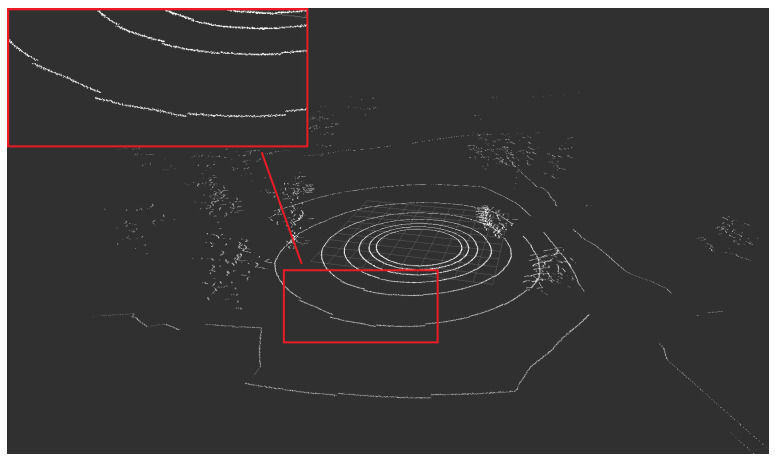
The rolling shutter effect visible on the point cloud obtained from a simulation.

We also present the graphical results in the form of a point cloud obtained in a simulation, in which the rolling shutter effect is visible. We decided to use the outdoor environment instead of the indoor one, because the simulation of this effect is more necessary for outdoor applications, in which a vehicle can reach a significant speed. It can be seen in Figure 15. When we compare the obtained effect with the one observed in a real-life setup (Figure 3), we can clearly see that the simulation can accurately mimic this behavior.

## 6. Conclusions

We have created the simulator which can be used to generate the realistic LiDAR 3D point clouds for the purpose of the SLAM algorithm evaluation. Its high accuracy, efficiency and the fact that it was adapted to simulate the rolling shutter effect that is a characteristic for actual devices that makes it a cost-effective alternative to using real-world setups and actual devices. Our framework can operate in real time, which also increases its usefulness in the area of SLAM algorithm evaluation. Due to the use of additional software elements created in the work, our simulation is compatible with ROS. These additional software elements enabled us to use the simulated and real-world data interchangeably in our experiments and can significantly facilitate the deployment of the novel SLAM algorithms.

When the simulated object is an actual, imperfect electronic device, it is crucial to know its characteristics and, equally important, the characteristics of errors generated by this device. To create an accurate simulation, it is vital to incorporate these errors in the model. The comparison of the point clouds obtained from an actual device and from a simulation shows that the adoption of the ‘noisy’ model of a simulation significantly increases its similarity to the one obtained in a corresponding real-life environment. Obviously, the accuracy and detailing of the simulation model have a crucial impact on the obtained results quality. For more complex environments, the difference between the errors of the SLAM algorithm calculated for real and simulated increases with the length of the test track. Because of that, for such tracks, it is better to use the simulated point clouds with additional noise to obtain accurate results of the SLAM evaluation.

The results obtained in the experiments have clearly shown that the rolling shutter effect should not be omitted in the simulation, as the error of some of the SLAM algorithms (e.g., Google Cartographer, which was used in the experiments) significantly increases with time when the effect is not present. The results obtained from the simulation without the rolling shutter effect suggest that the Google Cartographer SLAM algorithm performs poorly, though it is the simulation that does not reflect the true characteristics of data and gives misleading results. This shows that the data generated by the simulation should reflect the characteristics of the real world, on which the SLAM algorithm will run after deployment, to deliver an accurate verification in the testing process.

## Figures and Tables

**Table 1:** The parameters of and actual and simulated Velodyne VLP-16 device.

Parameter	Units	VLP-16	Simulation
Channels	-	16	16
Min–max vertical angle	degree	−15–15°	−15–15°
Horizontal samples	-	3600	3600
Min–max horizontal angle	degree	0–360°	0–360°
Horizontal FoV per simulation update	degree	2.4°	15°
Range	m	100 m	100 m
Range accuracy	m	0.03 m	0.03 m
Rotation rate	Hz	10	10
Mode	-	Strongest/last	Strongest

## Data Availability

Not applicable.
